# A comparative study of nano-selenium and selenite in alleviating drought stress in rice by regulating ion homeostasis

**DOI:** 10.1186/s12870-026-09022-7

**Published:** 2026-05-28

**Authors:** Kangkang Zhang, Yihua Wei, Xiaoyu Chang, Zhenghua Huang, Zaid Khan, Dandan Jin, Biaojin Zhang

**Affiliations:** 1 Institute for Quality & Safety and Standards of Agricultural Products Research, Jiangxi Academy of Agricultural Sciences, Nanchang, China; 2 Jiangxi Provincial Key Laboratory for Quality and Safety of Agricultural Products, Nanchang, China; 3 Jiangxi Provincial Selenium-Enriched Agricultural Industry Research Institute, Nanchang, China; 4 College of Architectural Engineering, Shenzhen Polytechnic University, Shenzhen, China

**Keywords:** Nano-selenium, Seed coating, Seed priming, Ionomic, Heavy metal detoxification, Rice

## Abstract

Drought stress is a major constraint on the safe and stable production of rice, and the use of selenium (Se) to enhance crop stress resistance has emerged as a promising agronomic strategy. To examine the effects of different selenium forms and application methods on the drought tolerance of rice, this study applied selenite and nano selenium (Nano-Se) through seed coating and seed priming, respectively. Drought stress was simulated under hydroponic conditions using Polyethylene glycol-6000 (PEG-6000). The results showed that Nano-Se treatment was more effective in mitigating growth inhibition, optimizing root architecture, maintaining photosynthetic efficiency, and improving leaf water status. Among the treatments, seed priming with 5 mg L^− 1^ Nano-Se produced the most pronounced benefits, performing significantly better than or comparable to the optimal selenite treatment. Notably, while enhancing selenium uptake, Nano-Se also reduced the accumulation of heavy metals (chromium, arsenic, cadmium, lead) in aboveground tissues under drought stress and helped maintain nutrient homeostasis. In contrast, high concentrations of selenite exhibited phytotoxic effects and potentially increased the risk of arsenic contamination. Overall, this study demonstrates that Nano-Se—characterized by low toxicity, slow-release behavior, and systemic regulatory functions—represents a highly promising novel formulation for improving drought resistance. These findings provide both theoretical insights and technical support for ensuring rice production safety and enhancing nutritional quality under complex environmental conditions.

## Introduction

Drought stress is one of the most critical abiotic factors limiting global agricultural production, severely affecting crop growth, development, physiological metabolism, and ultimately yield [1]. As a typical water-intensive crop, rice is particularly sensitive to water deficits throughout its entire growth cycle [2]. Particularly during the seedling and vegetative stages, water deficits often trigger physiological disorders and cause irreversible impacts on later yields [3]. The development of effective drought-resistant strategies has become a critical priority for ensuring global food security with accelerating global climate change and the increasing frequency of extreme drought events [4].

Among various drought-mitigation strategies, the use of trace or beneficial elements to activate plants’ inherent stress-resistance mechanisms has become an economically viable and environmentally sustainable agricultural approach [5]. Selenium (Se), recognized as a beneficial nutrient for plants, has been extensively demonstrated to enhance crop tolerance to abiotic stresses [6]. Numerous studies have shown that selenium application at appropriate concentrations significantly improves plant survival under drought conditions [7]. This effect is largely attributed to selenium’s ability to strengthen the antioxidant defense system. Selenium serves as an essential component of glutathione peroxidase, a key enzyme involved in reactive oxygen species (ROS) scavenging [8]. Experimental evidence further demonstrates that selenium treatment reduces malondialdehyde accumulation in maize leaves under drought stress, while increasing superoxide dismutase and peroxidase activities [9]. In addition, selenium contributes to drought tolerance by regulating endogenous hormone balance and promoting the accumulation of osmotic adjustment compounds. For instance, Nawaz et al. (2013) observed that selenium pretreatment increased proline content in rice leaves and simultaneously reduced abscisic acid levels, thereby helping maintain leaf relative water content [10].

However, the use of traditional inorganic selenium forms presents notable limitations. Selenite is readily adsorbed and immobilized by iron and aluminum oxides in soil, resulting in a bioavailability typically below 30% [11]. Moreover, selenium possesses a narrow effective concentration range, and excessive application can easily induce phytotoxicity [12]. These constraints greatly limit the efficient and safe application of conventional selenium fertilizers in agricultural production [13]. Recent advancements in nanotechnology have created new opportunities for improving selenium utilization efficiency [14]. Nanoscale selenium (Nano-Se) exhibits enhanced bioactivity and safety due to its nanoscale size, high specific surface area, and controlled-release characteristics [15]. Compared to traditional inorganic selenium, Nano-Se may exhibit distinct absorption and transportation within plants, potentially offering advantages in enhancing antioxidant capacity and alleviating abiotic stress. Mechanistic insights reveal that Nano-Se more effectively penetrates plant cell walls and is transported through the symplastic pathway, thereby substantially improving selenium utilization efficiency [16]. Existing research indicates that Nano-Se can effectively improve plant physiological status under drought [17], high temperature [18], and salt stress conditions [19], while also reducing the risk of harmful element accumulation to some extent [20–22].

In addition to the form of selenium applied, the method of application is also a critical factor influencing the effectiveness of selenium fertilization [23]. Seed treatment, as an early-stage intervention in the crop life cycle, offers advantages such as low cost, operational simplicity, and environmental sustainability [24]. Among these, seed priming and seed coating are considered the most promising approaches [25]. Seed priming initiates metabolic repair processes through controlled hydration, allowing seeds to absorb bioactive substances before germination and thereby providing seedlings with a form of pre-adaptation to stress [26]. Previous studies have shown that selenium-primed seeds exhibit increased antioxidant enzyme activity during germination, establishing a strong physiological basis for improved seedling stress tolerance [27]. In contrast, seed coating creates a protective layer on the seed surface that encapsulates active compounds, enabling their sustained release and offering prolonged protection during early seedling development [28]. Rocha et al. (2019) demonstrated that seed coating can extend the duration of active ingredient availability and increase utilization efficiency [29]. Although the above-mentioned technologies have been applied to some extent in agricultural production, direct comparisons of the synergistic effects and underlying mechanisms between different selenium sources and seed treatment methods remain lacking [30].

Based on the above observation, this study employed different concentrations of selenite and Nano-Se through seed priming and seed coating to systematically evaluate the effects of various selenium source–application method combinations on rice growth, physiological responses, and elemental uptake and transport under simulated drought stress conditions. The primary research objectives include: (1) Comparing the regulatory effects of different selenium sources in alleviating rice drought stress; (2) To evaluate the relative efficiency of seed priming versus seed coating in delivering different selenium sources and stimulating drought resistance responses; (3) To elucidate the potential mechanisms by which selenium treatments mitigate drought stress from the perspectives of ion homeostasis maintenance and physiological regulation. The findings provide a theoretical basis and technical support for the rational application of nano-selenium in drought-resistant rice production.

## Materials and methods

### Experimental materials and seed treatment

**Fig. 1:**
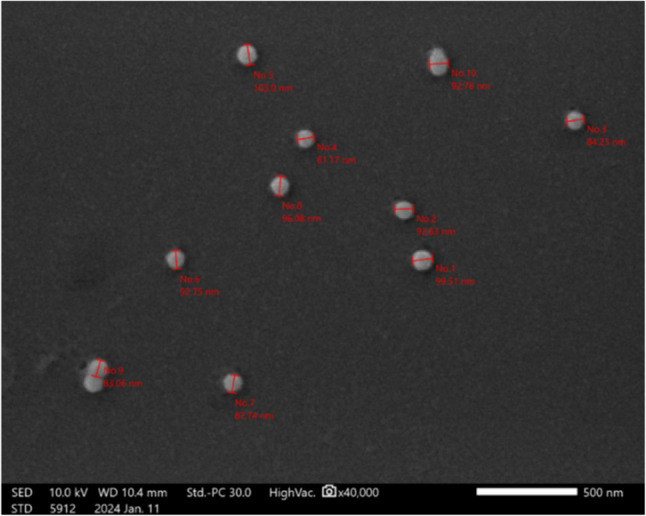
TEM image of Nano-Se. The white bar indicates 500 nm

This study utilized the conventional rice cultivar ‘Huanghuazhan’, supplied by the Plant Nutrition Team of the Jiangxi Academy of Agricultural Sciences (Nanchang, China), as the experimental material. Sodium selenite (SeO_3_^2−^, purity ≥ 97.0%) was obtained from Sinopharm Chemical Reagent Co., Ltd., while selenium nanoparticles (Nano-Se, purity ≥ 99.9%) were provided by Hengshui Gemai Trace Elements Co., Ltd. (Hengshui, China). Transmission electron microscopy (TEM) analysis showed that the Nano-Se particles possessed a uniform spherical morphology with an average diameter of approximately 91.30 nm (Fig. 1).

The concentration of selenium treatment in this study was determined by comprehensively referencing previous research on the biological effects of selenite and Nano-Se in rice and other crops [31, 32], combined with the preliminary screening results of our research group. Preliminary experiments showed that within the selected concentration range (5–20 mg L⁻¹ for seed priming, 25–100 mg L⁻¹ for coating), selenium treatment significantly affected rice seedling growth and stress resistance physiological processes, while no obvious toxic symptoms were observed, thus ensuring the safety and biological effectiveness of the experimental treatment.

The seed coating (SC) treatment was conducted as follows: Plump, uniformly sized rice seeds were selected, and 10 g were weighed for each treatment. The seed-coating agent was prepared at a seed-to-agent ratio of 1:800 (w/v). Subsequently, 0.3 mL of sodium selenite or Nano-Se solution at concentrations of 25, 50, or 100 mg L^− 1^ was added to the coating agent to prepare the working solution. The seeds and coating solution were then placed in a round-bottom container and thoroughly mixed to ensure uniform distribution of the coating material on the seed surface.

The seed priming (SP) treatment was performed as follows: Selected plump, uniformly sized rice seeds were surface-sterilized in 1% sodium hypochlorite solution for 15 min, rinsed three times with distilled water, and blotted dry with absorbent paper. The sterilized seeds were then immersed in sodium selenite or Nano-Se solutions at concentrations of 5, 10, or 20 mg L^− 1^ for 24 h at 25 °C, using a seed-to-solution ratio of 1:5 (w/v). After priming, the seeds were rinsed with distilled water for 2 min, blotted dry, and air-dried for 24 h to restore their original moisture content.

### Experimental design

The following treatment groups were established in this study: (1) untreated seedlings grown under normal moisture conditions (CK); (2) untreated seedlings subjected to drought stress (CK + DS). All remaining treatments were conducted under drought stress and included seed coating (SC) treatments using 25, 50, and 100 mg L^− 1^ selenite (SC+Se25, SC+Se50, SC+Se100) and equivalent concentrations of Nano-Se (SC+Nano25, SC+Nano50, SC+Nano100), as well as seed priming (SP) treatments using 5, 10, and 20 mg L^− 1^ selenite (SP+Se5, SP+Se10, SP+Se20) and corresponding concentrations of Nano-Se (SP+Nano5, SP+Nano10, SP+Nano20).

For the hydroponic experiment, treated and untreated rice seeds were placed on moist, sterile filter paper and germinated in darkness in an artificial climate chamber (HP250GS-C, Ruihua Instrument & Equipment Co., Ltd., Wuhan, China) set at 28 °C. After four days, the germinated seeds were transferred to hydroponic trays (127 × 87 × 114 mm) containing half-strength Hoagland nutrient solution. Seedlings were grown under the following controlled environmental conditions: a 14 h photoperiod, photosynthetic photon flux density (PPFD) of 150 µmol m⁻² s⁻¹, day/night temperatures of 30/25°C, and 70% relative humidity. On the third day after transplanting, the nutrient solution was replaced with full-strength Hoagland solution, and subsequent solution changes were performed every three days.

On the 19th day after transplanting, seedlings were transferred to Hoagland solution supplemented with 15% Polyethylene glycol-6000 (PEG-6000) to impose drought stress. During the stress period, water was replenished to maintain volume, but the nutrient solution was not replaced. After four days of drought treatment, leaf gas exchange parameters, root morphology, plant growth traits, and elemental uptake were measured sequentially. All treatments were performed with four biological replicates.

### Sampling and measurements

#### Plant growth indicators

From each replicate, three representative rice seedlings were selected, and their plant height and root length were measured individually. Shoots and roots were then harvested and separated, followed by sequential rinsing with running water and deionized water. Fresh weights were recorded immediately. The tissues were subsequently placed in an oven at 105 °C for 30 min to deactivate enzymes and halt metabolic activity, after which the temperature was reduced to 75 °C for drying to constant weight. After obtaining dry weights, samples were cut into small pieces for acid digestion. Detailed digestion procedures are provided in Sect. Quantification of nutritional and potentially toxic elements.

#### Root morphology

Root morphological analysis was performed using an Epson V800 scanner (Epson Seiko Epson Corporation, Suwa City, Nagano Prefecture, Japan). Fresh roots from six seedlings per treatment were scanned at a resolution of 300 dpi. Morphological parameters, including total root length, surface area, root volume, and number of root tips, were quantified using WinRHIZO 2024a software (Regent Instruments, Quebec City, QC, Canada).

#### Leaf relative water content (Leaf RWC)

Leaf RWC for both control and drought-stressed seedlings was assessed following the method of Lata et al. (2011) [33], with slight modifications. Fresh young leaves were collected from each replicate, cut into 1 cm segments, and weighed to obtain approximately 0.5 g fresh weight (FW). The samples were then immersed in distilled water at room temperature for 24 h. After removing surface moisture, turgid weight (TW) was recorded. The leaves were subsequently dried in an oven at 75 °C to constant dry weight (DW). RWC was calculated using the formula: $RWC\,\left(\%\right)=\left[\left(FW-DW\right)/\left(TW-DW\right)\right]\times100$.

#### SPAD and gas exchange parameters

SPAD values and photosynthetic parameters were measured on the second-to-last fully expanded leaf of each treatment. SPAD values were obtained using a SPAD-502 chlorophyll meter (Minolta Camera Co., Osaka, Japan). Gas exchange parameters, including net photosynthetic rate (*A*), transpiration rate (*E*), stomatal conductance (*g*_*sw*_), and intercellular CO₂ concentration (*C*_*i*_), were recorded using a LI-6800 portable photosynthesis system (LI-COR Inc., Lincoln, NE, USA). During measurements, the leaf chamber was maintained under the following conditions: CO₂ concentration of 400 µmol mol⁻¹, relative humidity of 60%, leaf temperature of 30 °C, PPFD of 1500 µmol m⁻² s⁻¹, and airflow rate of 600 µmol s⁻¹.

#### Quantification of nutritional and potentially toxic elements

Plant sample preparation and elemental analysis were performed following the method described by Paniz et al. (2018) [34], with minor modifications. Briefly, 0.1000 g of ground root tissue or 0.2000 g of ground shoot tissue (four replicates per treatment) was weighed into digestion tubes, and 6 mL of concentrated nitric acid was added. Samples were pre-digested at room temperature for 1 h, followed by digestion in a graphite block system (Beijing Labtech Instruments Co., Ltd., Beijing, China) at 60 °C for 1 h. The temperature was then increased to 140 °C and maintained until the solution became clear and the volume was reduced to approximately 1 mL. After cooling, the digest was diluted to 15 mL with ultrapure water. Element concentrations were determined using inductively coupled plasma-tandem mass spectrometry (ICP-MS/MS, NexION 5000G, PerkinElmer, Shelton, CT, USA) and inductively coupled plasma optical emission spectrometry (ICP-OES, Thermo Fisher Scientific, Bremen, Germany).

### Statistical analysis

Transfer factors (TF) for selenium (Se), chromium (Cr), arsenic (As), cadmium (Cd), and lead (Pb) were calculated as TF = C_shoot_ / C_root_, where C_shoot_ and C_root_ represent element concentrations in shoots and roots, respectively (expressed on a dry-weight basis). Total element accumulation in roots and shoots was calculated using the formulas $$\begin{aligned}T_{root}&=C_{root}\times\,root\,dry\,biomass\,and\,T_{shoot}\\&=C_{shoot}\times\,shoot\,dry\,biomass\end{aligned}$$.

Data analysis was conducted using Statistix 9.0 software. Differences among treatments were evaluated using one-way analysis of variance (ANOVA), followed by least significant difference (LSD) tests at *P* < 0.05. All figures were generated using the ggplot2 package in RStudio.

## Results

### Effects of seed treatment with Se on rice seedling growth

**Fig. 2:**
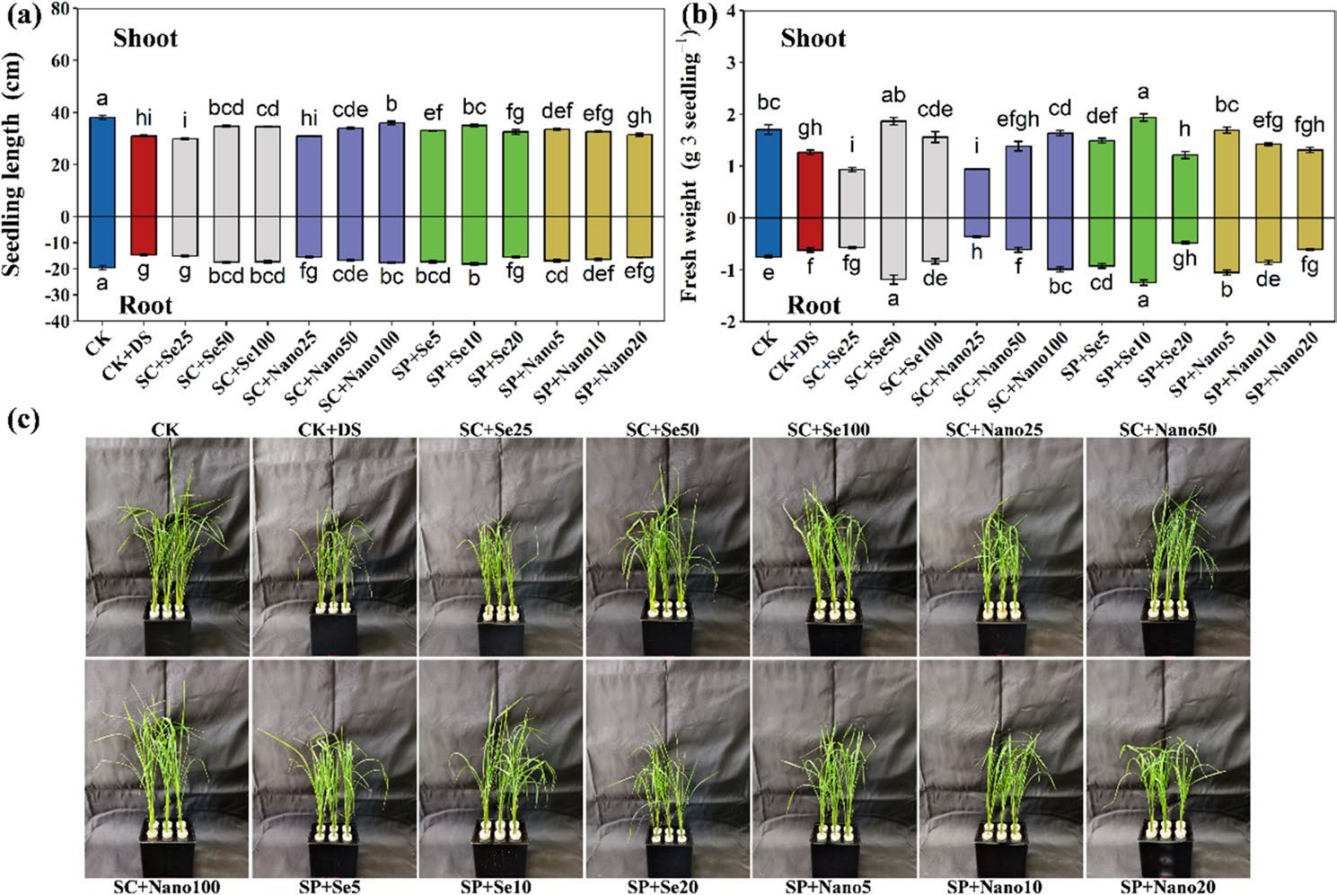
Growth parameters of 27-day-old rice seedlings after exogenous selenium seed treatment: (**a**) shoot length and root length; (**b**) shoot fresh weight and root fresh weight; (**c**) seedling phenotypic graph. Data represent the mean of four replicates; error bars indicate standard deviation. Different letters indicate significant differences among treatments at *p* < 0.05. Treatment descriptions: CK represents untreated seedlings under normal moisture conditions; CK + DS represents untreated seedlings under drought stress; SC+Se25, SC+Se50, SC+Se100 represent seed coating with 25, 50, or 100 mg L^− 1^ selenite, respectively, under drought stress; SC+Nano25, SC+Nano50, SC+Nano100 represent seed coating with 25, 50, and 100 mg L^− 1^ nano-selenium under drought stress, respectively; SP+Se5, SP+Se10, and SP+Se20 represent seed priming with 5, 10, and 20 mg L^− 1^ selenite, respectively, under drought stress; SP+Nano5, SP+Nano10, and SP+Nano20 represent seed priming with 5, 10, and 20 mg L^− 1^ Nano-selenium, respectively, under drought stress

Drought stress markedly inhibited the growth of rice seedlings, whereas appropriate seed treatment methods and concentrations of exogenous selenium significantly alleviated this inhibition (Fig. 2a-c). In seed coating treatments, increasing selenite concentrations first enhanced then suppressed shoot length, root length, and seedling fresh weight, with SC+Se50 showing the most pronounced promotive effect. For nano-selenium, growth promotion increased steadily with concentration, and SC+Nano100 produced the best results (Fig. 2a, b). In seed-priming treatments, the effect of selenite followed a pattern similar to that observed in coating treatments, with SP+Se10 being the most effective. In contrast, nano-selenium displayed diminishing growth-promoting effects with increasing concentrations, and SP+Nano5 yielded the greatest improvement (Fig. 2a, b). Overall, SC+Se50 and SP+Se10 were the most effective treatments in mitigating drought-induced inhibition of seedling growth, with no significant differences between them. SC+Nano100 and SP+Nano5 were the next most effective treatments. No significant differences were observed among different selenium application methods or sources with respect to improving seedling drought tolerance.

### Effects of seed treatment with Se on root morphology of rice seedlings

**Fig. 3:**
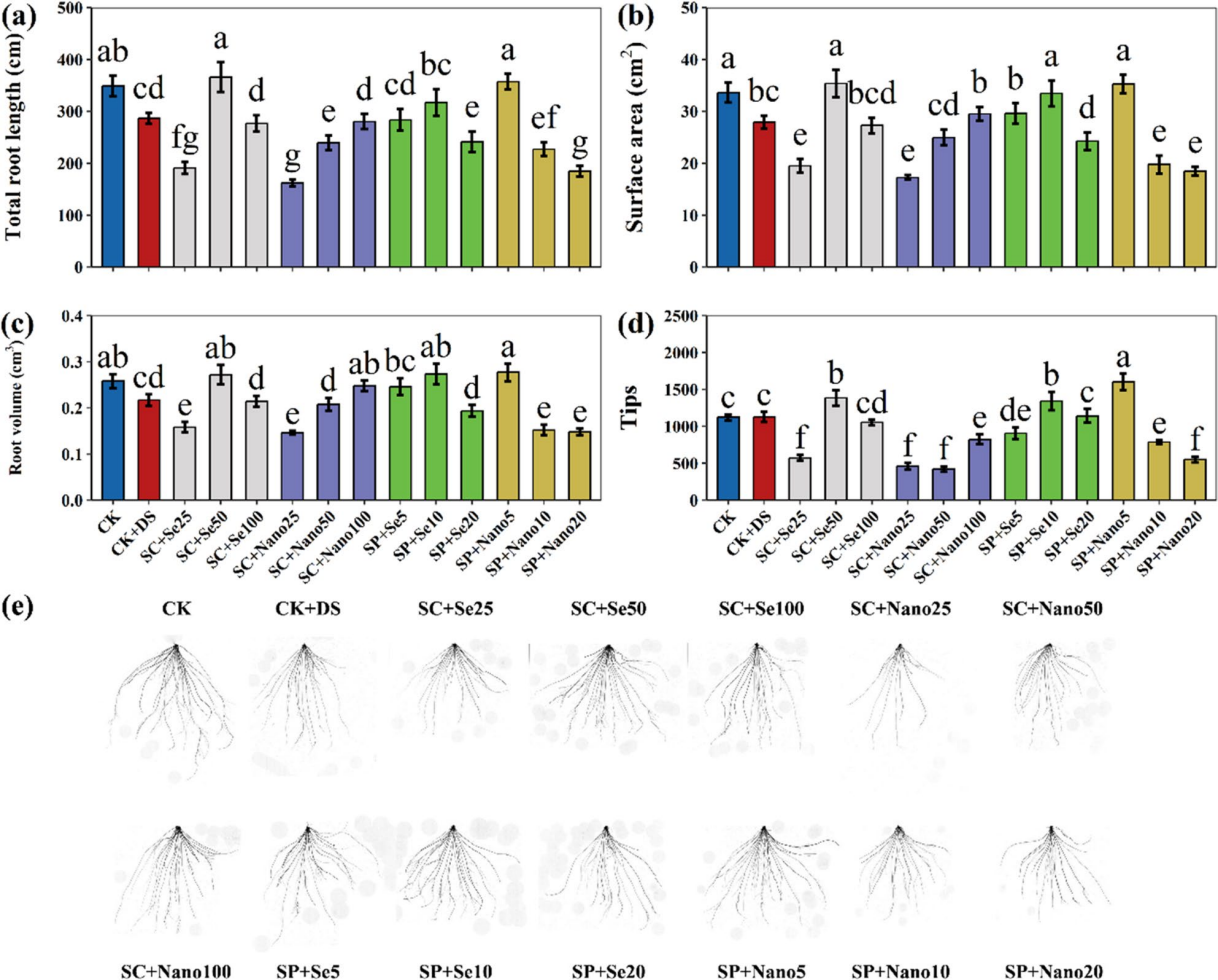
Root morphological parameters of 27-day-old rice seedlings after exogenous selenium seed treatment: (**a**) total root length; (**b**) surface area; (**c**) root volume; (**d**) tips; (**e**) root phenotypic graph. Data represent the mean of four replicates; error bars indicate standard deviation. Different letters indicate significant differences among treatments at *p* < 0.05. Treatment descriptions: CK represents untreated seedlings under normal moisture conditions; CK + DS represents untreated seedlings under drought stress; SC+Se25, SC+Se50, SC+Se100 represent seed coating with 25, 50, or 100 mg L^− 1^ selenite, respectively, under drought stress; SC+Nano25, SC+Nano50, SC+Nano100 represent seed coating with 25, 50, and 100 mg L^− 1^ Nano-selenium under drought stress, respectively; SP+Se5, SP+Se10, and SP+Se20 represent seed priming with 5, 10, and 20 mg L^− 1^ selenite, respectively, under drought stress; SP+Nano5, SP+Nano10, and SP+Nano20 represent seed priming with 5, 10, and 20 mg L^− 1^ Nano-selenium, respectively, under drought stress

Under drought stress, root development in untreated seedlings was severely restricted (Fig. 3a-e). In contrast, suitable exogenous selenium treatments substantially alleviated this inhibition. In seed coating treatments, root morphological parameters—including total root length, surface area, root volume, and root tips—initially increased and then decreased with rising selenite concentrations, with SC+Se50 showing the strongest improvement (Fig. 3a-e). Nano-selenium consistently enhanced root growth at higher concentrations, with SC+Nano100 being the most effective; however, only this treatment produced a significantly higher root volume compared with CK + DS (Fig. 3c). In seed-priming treatments, the effects of selenite mirrored those observed with coating, with SP+Se10 demonstrating the greatest improvement. Nano-selenium showed diminishing effects with increasing concentrations, and SP+Nano5 exhibited the strongest promotion of root development (Fig. 3a, b). Overall, SP+Nano5 provided the greatest alleviation of drought-induced suppression of root growth, followed by SC+Se50 and SP+Se10.

### Effects of seed treatment with Se on leaf RWC, SPAD, and gas exchange parameters

**Fig. 4:**
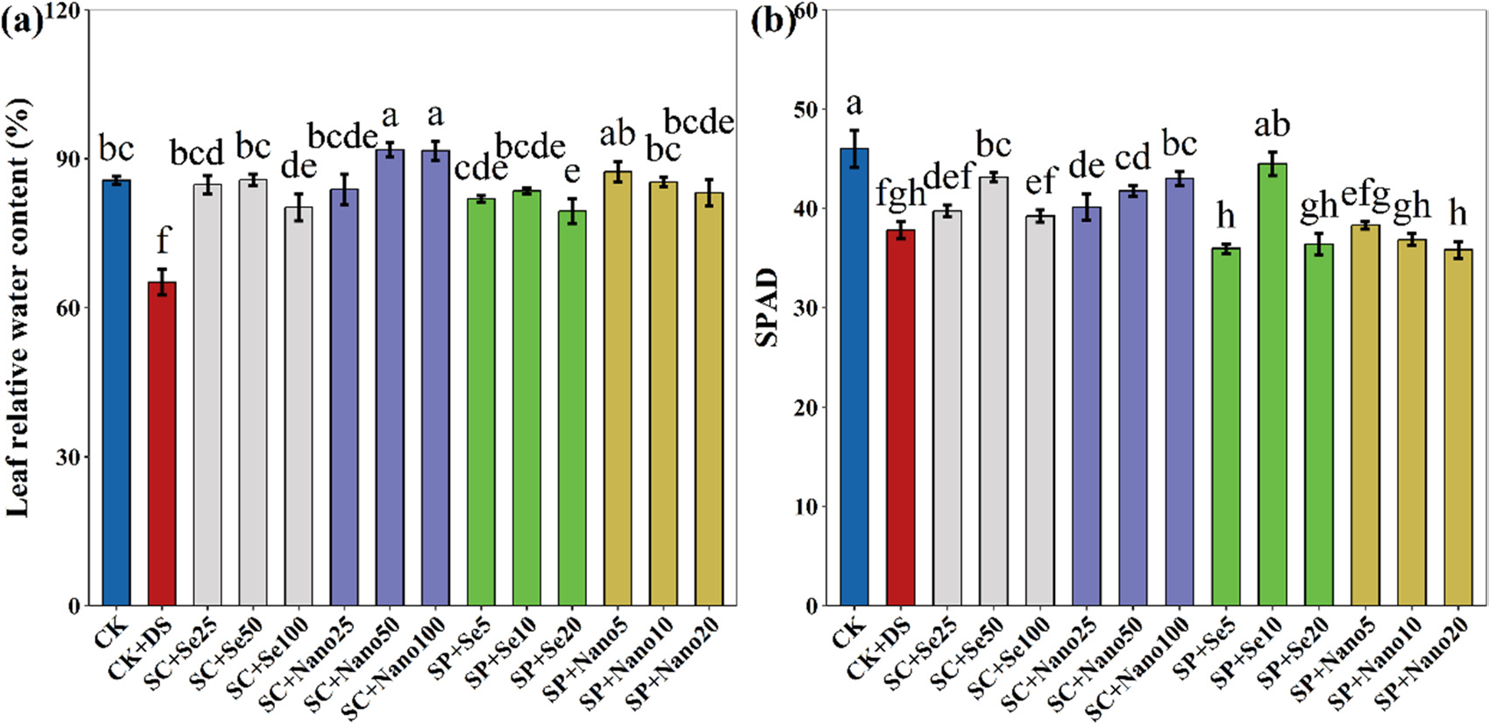
Relative leaf water content (**a**) and SPAD (**b**) of 27-day-old rice seedlings after exogenous selenium seed treatment. Data represent the mean of four replicates; error bars indicate standard deviation. Different letters indicate significant differences among treatments at *p* < 0.05. Treatment descriptions: CK represents untreated seedlings under normal moisture conditions; CK + DS represents untreated seedlings under drought stress; SC+Se25, SC+Se50, SC+Se100 represent seed coating with 25, 50, or 100 mg L^− 1^ selenite, respectively, under drought stress; SC+Nano25, SC+Nano50, SC+Nano100 represent seed coating with 25, 50, and 100 mg L^− 1^ Nano-selenium under drought stress, respectively; SP+Se5, SP+Se10, and SP+Se20 represent seed priming with 5, 10, and 20 mg L^− 1^ selenite, respectively, under drought stress; SP+Nano5, SP+Nano10, and SP+Nano20 represent seed priming with 5, 10, and 20 mg L^− 1^ Nano-selenium, respectively, under drought stress

Regardless of the form of exogenous selenium, the response patterns of leaf RWC and SPAD values under drought stress were consistent with overall seedling growth performance (Fig. 4). Drought stress caused a significant reduction in leaf RWC, whereas exogenous selenium treatments effectively mitigated this decline, with all treatments showing significantly higher RWC than CK + DS (Fig. 4a). Specifically, in coating treatments, selenite and Nano-selenium increased RWC by 23.2%-31.8% and 28.7%-41.2%, respectively. In priming treatments, the corresponding increases were 22.1%-28.3% and 27.8%-34.3%. For SPAD values, significant increases relative to CK + DS were observed only in SC+Se50, SP+Se10, and all Nano-selenium coating treatments, with improvements of 14.3%, 18.0%, and 6.1%-13.8%, respectively (Fig. 4b). Overall, SC+Nano100, SC+Se50, and SP+Se10 were the most effective treatments in alleviating drought-induced reductions in leaf water status and chlorophyll content.

**Fig. 5:**
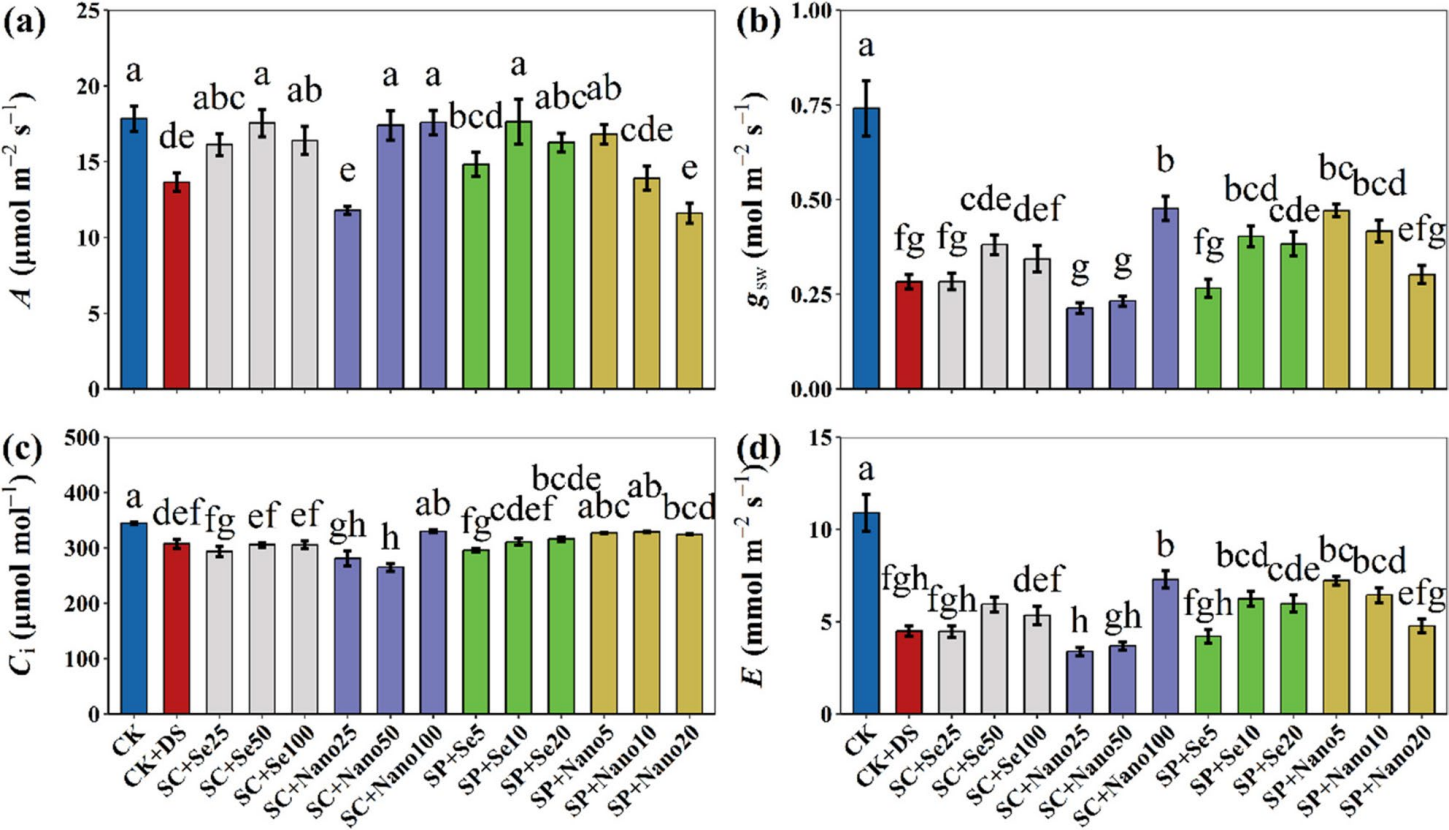
*A* (**a**), *g*_*sw*_ (**b**), *Ci* (**c**), and *E* (**d**) of 27-day-old rice seedlings after exogenous selenium seed treatment. Data represent the mean of four replicates; error bars indicate standard deviation. Different letters indicate significant differences among treatments at *p* < 0.05. Treatment descriptions: CK represents untreated seedlings under normal moisture conditions; CK + DS represents untreated seedlings under drought stress; SC+Se25, SC+Se50, SC+Se100 represent seed coating with 25, 50, or 100 mg L^− 1^ selenite, respectively, under drought stress; SC+Nano25, SC+Nano50, SC+Nano100 represent seed coating with 25, 50, and 100 mg L^− 1^ Nano-selenium under drought stress, respectively; SP+Se5, SP+Se10, and SP+Se20 represent seed priming with 5, 10, and 20 mg L^− 1^ selenite, respectively, under drought stress; SP+Nano5, SP+Nano10, and SP+Nano20 represent seed priming with 5, 10, and 20 mg L^− 1^ Nano-selenium, respectively, under drought stress

Drought stress significantly decreased all measured gas exchange parameters, including *A*, *g*_*sw*_, *Ci*, and *E*, whereas exogenous selenium application at optimal modes and concentrations resulted in significantly improved values relative to the drought-stressed control (Fig. 5a-d). The response patterns largely paralleled those observed for seedling growth. In coating treatments, all treatments except SC+Nano25 significantly increased *A*, with SC+Se50 and SC+Nano100 showing the greatest enhancements (28.5% and 28.8%, respectively) (Fig. 5a). In priming treatments, only SP+Se10, SP+Se20, and SP+Nano5 significantly increased *A*, with increases of 29.1%, 19.1%, and 23.1%, respectively. For *g*_*sw*_, significant increases were detected only in SC+Se50 and SC+Nano100 among coating treatments. In priming treatments, all treatments except SP+Se5 and SP+Nano20 significantly improved *g*_*sw*_, with SP+Se10 and SP+Nano5 showing the greatest effects (Fig. 5b). *Ci* increased significantly only in SC+Nano100, SP+Nano5, and SP+Nano10 (Fig. 5c). The trend in *E* closely mirrored that of *g*_*sw*_ (Fig. 5d). Overall, SC+Nano100 and SP+Nano5 were the most effective treatments in enhancing photosynthetic performance under drought stress.

### Effects of seed treatment with Se on elemental uptake and accumulation

#### Se uptake and accumulation

**Fig. 6:**
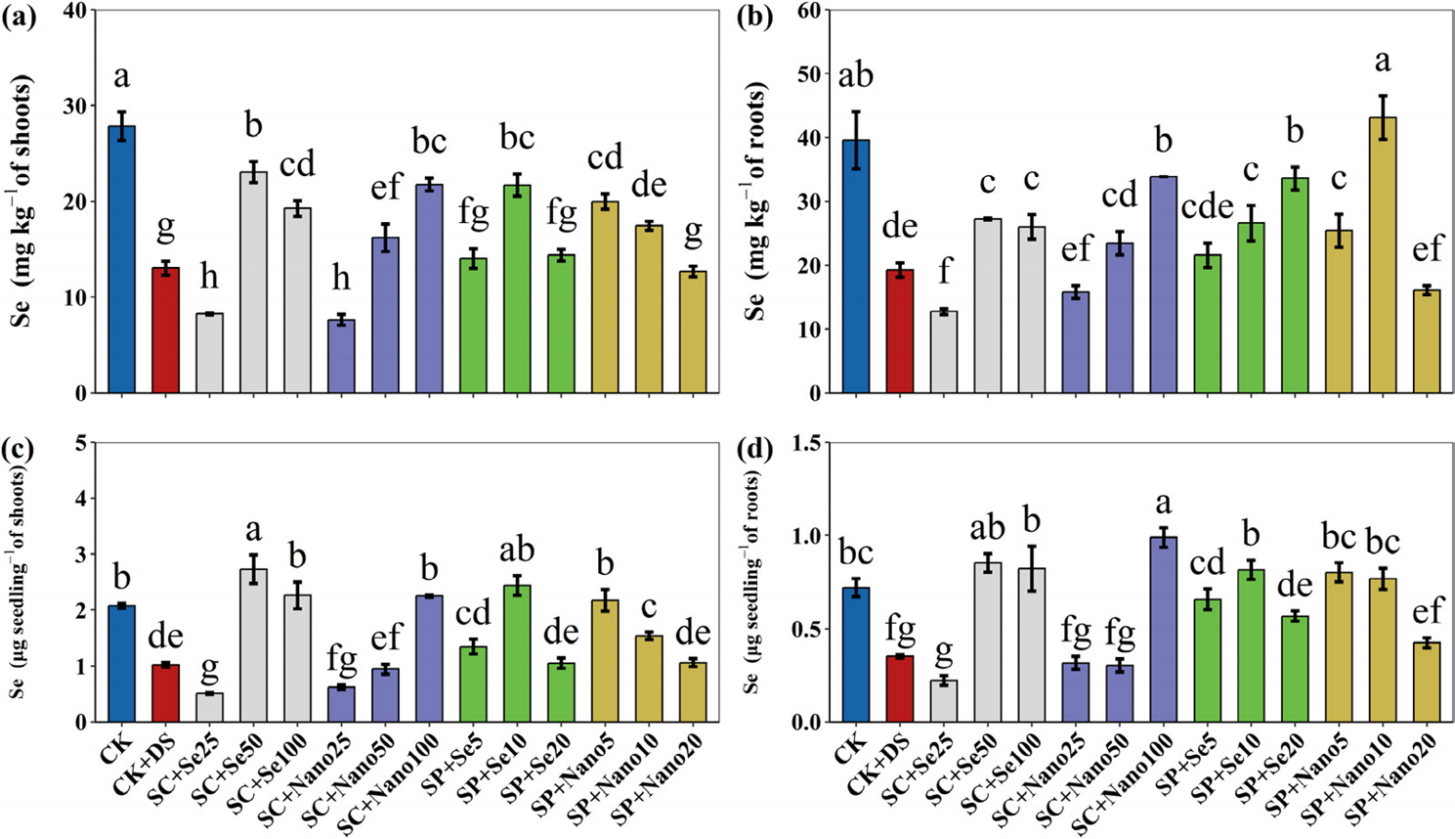
The uptake (**a**, **b**) and accumulation (**c**, **d**) of Se concentrations in the shoots (**a**, **c**) and roots (**b**, **d**) of 27-day-old rice seedlings after exogenous selenium seed treatment. Data represent the mean of four replicates; error bars indicate standard deviation. Different letters indicate significant differences among treatments at *p* < 0.05. Treatment descriptions: CK represents untreated seedlings under normal moisture conditions; CK + DS represents untreated seedlings under drought stress; SC+Se25, SC+Se50, SC+Se100 represent seed coating with 25, 50, or 100 mg L^− 1^ selenite, respectively, under drought stress; SC+Nano25, SC+Nano50, SC+Nano100 represent seed coating with 25, 50, and 100 mg L^− 1^ Nano-selenium under drought stress, respectively; SP+Se5, SP+Se10, and SP+Se20 represent seed priming with 5, 10, and 20 mg L^− 1^ selenite, respectively, under drought stress; SP+Nano5, SP+Nano10, and SP+Nano20 represent seed priming with 5, 10, and 20 mg L^− 1^ Nano-selenium, respectively, under drought stress

Drought stress caused substantial reductions in Se concentrations in both shoots and roots of untreated rice seedlings, with decreases of 53.2% and 58.6%, respectively (Fig. 6a, b). In contrast, appropriate exogenous Se treatments effectively enhanced Se uptake. In seed coating treatments, increasing selenite concentrations produced a pattern of initially rising and then declining Se content in both shoots and roots. SC+Se50 and SC+Se100 exhibited the strongest effects, increasing shoot Se concentrations by 76.8% and 47.6%, respectively, and root Se concentrations by 41.5% and 35.0%. Nano-Se consistently promoted Se uptake with increasing concentration, with SC+Nano100 showing the greatest enhancement—raising Se content in shoots and roots by 66.9% and 75.8%, respectively (Fig. 6a, b). In seed priming treatments, shoot Se concentrations initially increased and then declined with rising selenite concentrations, with SP+Se10 achieving the highest increase (66.3%). Root Se concentrations, however, showed a continuous upward trend, peaking at 74.4% in the SP+Se20 treatment. For Nano-Se priming, shoot Se content decreased with increasing concentration, with SP+Nano5 being the most effective (a 53.2% increase). In roots, Nano-Se priming produced an initial increase followed by a decline, with SP+Nano10 resulting in the greatest increase (123.9%).

The patterns of total Se accumulation in seedling shoots and roots generally paralleled those of Se concentration, except for root accumulation in priming treatments. Drought stress significantly reduced total Se accumulation in both tissues, with decreases of 103.9% in shoots and 51.4% in roots (Fig. 6c, d). Among coating treatments, SC+Se50 and SC+Se100 increased total shoot Se accumulation by 121.6%-167.6% and root accumulation by 134.3%-142.9%. SC+Nano100 also markedly enhanced Se accumulation, increasing shoot and root totals by 120.6% and 182.9%, respectively (Fig. 6c, d). In priming treatments, total Se accumulation in selenite-treated groups showed an increase followed by a decline with rising concentrations. SP+Se10 provided the greatest enhancement, significantly increasing shoot and root accumulation by 139.2% and 134.3%, respectively. For Nano-Se, treatment efficacy decreased with increasing concentration, with SP+Nano5 demonstrating the strongest effect, increasing shoot and root accumulation by 113.7% and 128.6%, respectively. Overall, SC+Se50 and SP+Se10 were the most effective treatments for enhancing Se uptake and accumulation in rice seedlings, followed by SC+Nano100 and SP+Nano5.

#### Cr, As, Cd, and Pb uptake and accumulation

**Fig. 7:**
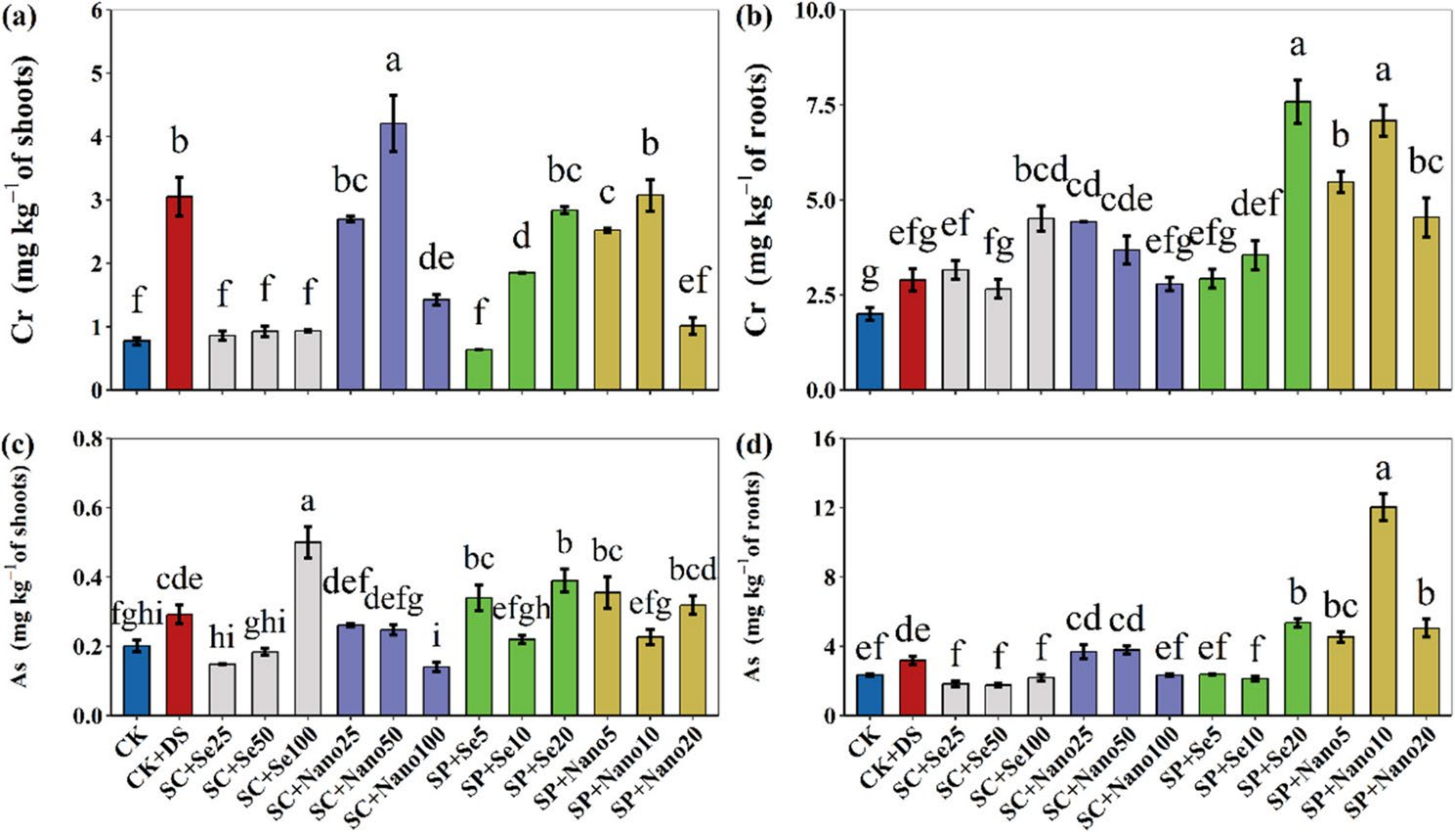
Concentrations of Cr (**a**, **b**) and As (**c**, **d**) in the shoots (**a**, **c**) and roots (**b**, **d**) of 27-day-old rice seedlings after exogenous selenium seed treatment. Data represent the mean of four replicates; error bars indicate standard deviation. Different letters indicate significant differences among treatments at *p* < 0.05. Treatment descriptions: CK represents untreated seedlings under normal moisture conditions; CK + DS represents untreated seedlings under drought stress; SC+Se25, SC+Se50, SC+Se100 represent seed coating with 25, 50, or 100 mg L^− 1^ selenite, respectively, under drought stress; SC+Nano25, SC+Nano50, SC+Nano100 represent seed coating with 25, 50, and 100 mg L^− 1^ Nano-selenium under drought stress, respectively; SP+Se5, SP+Se10, and SP+Se20 represent seed priming with 5, 10, and 20 mg L^− 1^ selenite, respectively, under drought stress; SP+Nano5, SP+Nano10, and SP+Nano20 represent seed priming with 5, 10, and 20 mg L^− 1^ Nano-selenium, respectively, under drought stress

Drought stress markedly increased Cr and As concentrations in the aboveground tissues of untreated rice seedlings, with increases of 291.0% and 45.3%, respectively. Although Cr and As concentrations in roots also increased by 45.0% and 35.7%, these changes were not statistically significant (Fig. 7a, b). Appropriate exogenous Se treatments effectively suppressed Cr and As uptake. In seed coating treatments, low selenite concentrations (SC+Se25, SC+Se50) significantly reduced shoot Cr, shoot As, and root As concentrations by 69.5%-71.8%, 37.0%-49.3%, and 42.6%-44.8%, respectively, compared to CK + DS (Fig. 7a, c, d). In contrast, high-concentration selenite (SC+Se100) significantly increased root Cr and shoot As concentrations by 55.5% and 71.2%, respectively (Fig. 7b, c). Among Nano-Se treatments, SC+Nano100 significantly decreased shoot Cr and As concentrations by 53.1% and 52.1%, respectively (Fig. 7a, c). In seed priming treatments, low selenite concentrations (SP+Se5, SP+Se10) significantly reduced shoot Cr concentrations by 39.3%-79.0%, and SP+Se10 further decreased root As concentration by 32.9%. However, the high selenite concentration (SP+Se20) significantly increased root Cr, shoot As, and root As concentrations by 161.4%, 33.2%, and 68.3%, respectively (Fig. 7b-d). For Nano-Se priming, SP+Nano5 and SP+Nano20 significantly reduced shoot Cr concentrations by 17.4%-66.6% (Fig. 7a), but all Nano-Se priming treatments markedly increased root Cr and As concentrations by 56.6%-144.5% and 42.3%-277.4%, respectively (Fig. 7b, d).

The patterns of total Cr and As accumulation generally mirrored those of their tissue concentrations. Except for Cr accumulation in roots, drought stress significantly increased total Cr and As accumulation in rice tissues (Table 1). Compared to CK + DS, low-concentration selenite treatments (SC+Se25, SP+Se5) and high-concentration Nano-Se (SC+Nano100) significantly reduced total Cr and As accumulation in shoots. Among coating treatments, high-concentration Se applications (SC+Se100, SC+Nano100) significantly increased total Cr accumulation in roots. In priming treatments, high-concentration selenite (SP+Se20) and all Nano-Se treatments significantly increased total Cr and As accumulation in roots (Table 1). Overall, SC+Se50 and SC+Nano100 were the most effective treatments for reducing Cr and As uptake and accumulation in rice seedlings.

**Table 1:** Accumulation of the Cr, As, Cd, and Pb in the shoots and roots of 27-day-old rice seedlings after exogenous selenium seed treatment

Tissue	Treatment	Cr (µg seedling^− 1^)	As (µg seedling^− 1^)	Cd (µg seedling^− 1^)	Pb (µg seedling^− 1^)
Shoot	CK	0.058 ± 0.005 e	0.0149 ± 0.0012 ghi	0.0015 ± 0.0000 e	0.0052 ± 0.0003 f
	CK + DS	0.238 ± 0.027 ab	0.0228 ± 0.0023 def	0.0028 ± 0.0002 ab	0.0114 ± 0.0005 a
	SC+Se25	0.054 ± 0.011 e	0.0092 ± 0.0005 i	0.0022 ± 0.0001 de	0.0038 ± 0.0005 g
	SC+Se50	0.108 ± 0.006 cd	0.0217 ± 0.0032 ef	0.0024 ± 0.0001 cd	0.0086 ± 0.0007 bc
	SC+Se100	0.110 ± 0.024 cd	0.0580 ± 0.0084 a	0.0029 ± 0.0002 ab	0.0074 ± 0.0009 de
	SC+Nano25	0.220 ± 0.011 b	0.0213 ± 0.0002 efg	0.0033 ± 0.0003 a	0.0091 ± 0.0004 b
	SC+Nano50	0.245 ± 0.042 ab	0.0144 ± 0.0011 hi	0.0020 ± 0.0004 de	0.0044 ± 0.0005 fg
	SC+Nano100	0.148 ± 0.012 c	0.0144 ± 0.0019 hi	0.0020 ± 0.0002 de	0.0045 ± 0.0008 f
	SP+Se5	0.061 ± 0.003 e	0.0323 ± 0.0053 bc	0.0025 ± 0.0003 bc	0.0109 ± 0.0006 a
	SP+Se10	0.208 ± 0.014 b	0.0246 ± 0.0010 def	0.0021 ± 0.0001 de	0.0113 ± 0.0000 a
	SP+Se20	0.209 ± 0.042 b	0.0281 ± 0.0009 cd	0.0022 ± 0.0002 de	0.0046 ± 0.0007 f
	SP+Nano5	0.274 ± 0.029 a	0.0380 ± 0.0049 b	0.0019 ± 0.0001 de	0.0051 ± 0.0003 f
	SP+Nano10	0.273 ± 0.057 a	0.0201 ± 0.0037 fgh	0.0021 ± 0.0000 de	0.0068 ± 0.0004 e
	SP+Nano20	0.084 ± 0.018 de	0.0266 ± 0.0045 cde	0.0021 ± 0.0003 de	0.0080 ± 0.0009 cd
Root	CK	0.037 ± 0.007 f	0.0430 ± 0.0023 hi	0.0019 ± 0.0001 e	0.0198 ± 0.0006 g
	CK + DS	0.053 ± 0.006 f	0.0581 ± 0.0046 efg	0.0045 ± 0.0002 a	0.0304 ± 0.0005 f
	SC+Se25	0.054 ± 0.003 f	0.0314 ± 0.0017 i	0.0037 ± 0.0005 bcd	0.0307 ± 0.0040 ef
	SC+Se50	0.082 ± 0.007 e	0.0548 ± 0.0049 fgh	0.0042 ± 0.0001 ab	0.0365 ± 0.0024 cdef
	SC+Se100	0.141 ± 0.025 b	0.0691 ± 0.0177 de	0.0031 ± 0.0008 cd	0.0393 ± 0.0099 bcde
	SC+Nano25	0.090 ± 0.025 de	0.0728 ± 0.0082 d	0.0032 ± 0.0009 bcd	0.0435 ± 0.0114 abcd
	SC+Nano50	0.047 ± 0.004 f	0.0485 ± 0.0009 gh	0.0027 ± 0.0001 cd	0.0297 ± 0.0014 f
	SC+Nano100	0.082 ± 0.013 e	0.0680 ± 0.0032 def	0.0026 ± 0.0001 cd	0.0353 ± 0.0020 def
	SP+Se5	0.089 ± 0.011 de	0.0729 ± 0.0005 d	0.0035 ± 0.0005 abc	0.0449 ± 0.0037 abc
	SP+Se10	0.109 ± 0.008 cd	0.0661 ± 0.0064 def	0.0030 ± 0.0003 cd	0.0306 ± 0.0029 ef
	SP+Se20	0.129 ± 0.021 bc	0.0905 ± 0.0016 c	0.0030 ± 0.0000 cd	0.0452 ± 0.0015 abc
	SP+Nano5	0.174 ± 0.017 a	0.1442 ± 0.0165 b	0.0037 ± 0.0004 abc	0.0458 ± 0.0068 ab
	SP+Nano10	0.126 ± 0.014 bc	0.2142 ± 0.0163 a	0.0027 ± 0.0002 de	0.0485 ± 0.0052 a
	SP+Nano20	0.118 ± 0.011 bc	0.1315 ± 0.0069 b	0.0030 ± 0.0002 cd	0.0426 ± 0.0051 abcd

The data are presented as the mean ± standard deviation (*n* = 4). Different letters indicate significant differences among treatments at *p* < 0.05

**Fig. 8:**
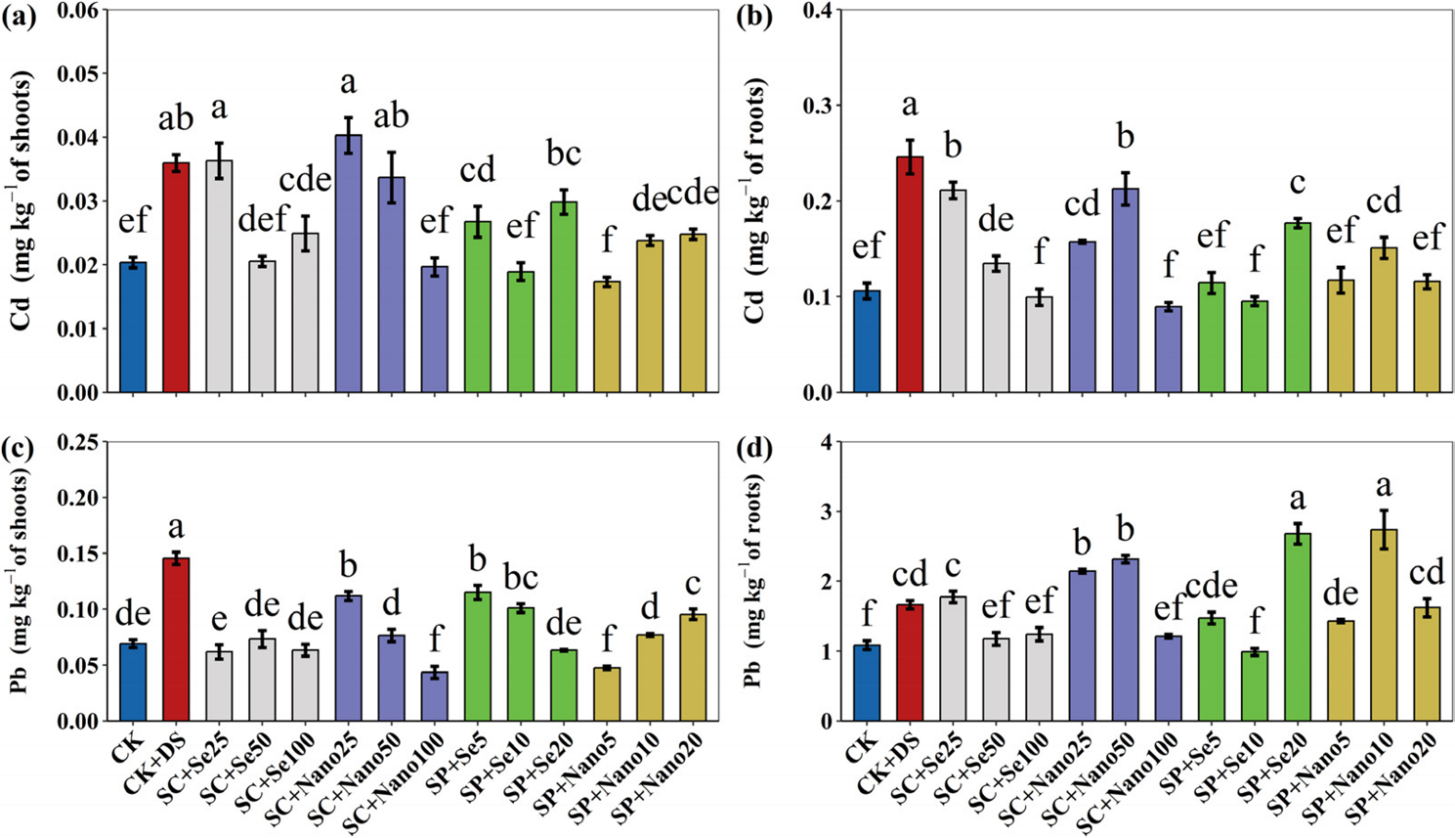
Concentrations of Cd (**a**, **b**) and Pb (**c**, **d**) in the shoots (**a**, **c**) and roots (**b**, **d**) of 27-day-old rice seedlings after exogenous selenium seed treatment. Data represent the mean of four replicates; error bars indicate standard deviation. Different letters indicate significant differences among treatments at *p* < 0.05. Treatment descriptions: CK represents untreated seedlings under normal moisture conditions; CK + DS represents untreated seedlings under drought stress; SC+Se25, SC+Se50, SC+Se100 represent seed coating with 25, 50, or 100 mg L^− 1^ selenite, respectively, under drought stress; SC+Nano25, SC+Nano50, SC+Nano100 represent seed coating with 25, 50, and 100 mg L^− 1^ Nano-selenium under drought stress, respectively; SP+Se5, SP+Se10, and SP+Se20 represent seed priming with 5, 10, and 20 mg L^− 1^ selenite, respectively, under drought stress; SP+Nano5, SP+Nano10, and SP+Nano20 represent seed priming with 5, 10, and 20 mg L^− 1^ Nano-selenium, respectively, under drought stress

Drought stress markedly increased Cd and Pb concentrations in untreated rice seedlings, with shoot Cd and Pb levels rising by 80.0% and 111.6%, respectively, and root Cd and Pb levels increasing by 132.1% and 53.2% (Fig. 8a-d). Appropriate exogenous Se treatments effectively mitigated Cd and Pb uptake. Among seed coating treatments, SC+Se50 and SC+Se100 reduced Cd concentrations in shoots and roots by 30.6%-41.7% and 45.1%-59.8%, respectively, while Pb concentrations decreased by 50.0%-56.8% and 25.7%-29.3%, respectively. All Nano-Se coating treatments significantly reduced root Cd and shoot Pb concentrations by 13.4%-63.4% and 23.3%-69.9%, respectively (Fig. 8b, c). Notably, SC+Nano100 decreased shoot and root Cd concentrations by 44.4% and 63.4%, respectively, and reduced shoot and root Pb concentrations by 69.9% and 27.5%. In seed priming treatments, all selenite treatments except SP+Se20 reduced Cd and Pb uptake to varying degrees. SP+Se10 exhibited the strongest effect, decreasing Cd concentrations in shoots and roots by 47.2% and 61.4%, respectively, and Pb concentrations by 30.8% and 40.7% (Fig. 8a-d). For Nano-Se priming, all treatments significantly decreased Cd and Pb concentrations—except for root Pb—with reductions in shoot and root Cd of 30.6%-52.8% and 38.6%-52.8%, respectively, and reductions in shoot Pb of 34.9%-67.8%. SP+Nano5 showed the most pronounced effect among priming treatments.

Drought stress also significantly increased total Cd and Pb accumulation in seedling tissues (Table 1). For shoots, SC+Se25, SC+Se50, SC+Nano50, SC+Nano100, SP+Se10, and all Nano-Se priming treatments significantly reduced total Cd and Pb accumulation. In roots, however, high selenite concentrations and all Nano-Se treatments significantly increased total Cd and Pb accumulation. Overall, SC+Se50, SC+Nano100, SP+Se10, and SP+Nano5 were the most effective treatments in inhibiting Cd and Pb uptake and accumulation in rice seedlings.

#### Macro- and micronutrient uptake and accumulation

Regarding macronutrient uptake, compared with CK, the CK + DS treatment significantly increased only potassium (K) and magnesium (Mg) concentrations in roots (Table 2). For aboveground tissues, both SC+Nano100 and SP+Se10 significantly enhanced K, calcium (Ca), and Mg concentrations (except that the Ca increase in SP+Se10 was not significant), whereas SC+Se50 and SP+Nano5 showed moderate but non-significant increases. In roots, all exogenous Se treatments—except SC+Nano25, SC+Nano50, SP+Se20, and SP+Nano20—significantly increased K concentration. Moreover, K, Ca, and Mg concentrations under SC+Se50, SC+Nano100, SP+Se10, and SP+Nano5 were comparable to or slightly higher than those under CK + DS, although the differences were not significant.

**Table 2:** Concentrations of the macro and micronutrients in the shoots and roots of 27-day-old rice seedlings after exogenous selenium seed treatment

Tissue	Treatment	K (g kg^− 1^)	Ca (g kg^− 1^)	Mg (g kg^− 1^)	Mn (g kg^− 1^)	Fe (g kg^− 1^)	Cu (mg kg^− 1^)
Shoot	CK	33.3 ± 33.3 f	3.67 ± 0.13 cde	3.90 ± 0.09 d	1.245 ± 1.245 d	0.128 ± 0.128 b	28.6 ± 1.3 bc
	CK + DS	32.2 ± 32.2 ef	3.86 ± 0.43 bcd	4.00 ± 0.68 cd	1.100 ± 1.100 e	0.115 ± 0.115 bcd	26.6 ± 2.1 cd
	SC+Se25	36.1 ± 36.1 bcde	3.24 ± 0.27 e	3.56 ± 0.32 d	0.793 ± 0.793 i	0.095 ± 0.095 de	23.2 ± 2.3 fgh
	SC+Se50	33.3 ± 33.3 def	3.90 ± 0.02 bcd	4.00 ± 0.18 cd	1.452 ± 1.452 bc	0.113 ± 0.113 bcd	27.0 ± 1.5 cd
	SC+Se100	39.3 ± 39.3 a	3.33 ± 0.23 e	3.86 ± 0.27 d	0.945 ± 0.945 fgh	0.099 ± 0.099 d	25.4 ± 1.3 def
	SC+Nano25	36.4 ± 36.4 abcd	3.47 ± 0.36 de	3.61 ± 0.36 d	1.000 ± 1.000 efgh	0.124 ± 0.124 bc	22.9 ± 0.3 gh
	SC+Nano50	34.8 ± 34.8 cdef	4.24 ± 0.56 b	4.64 ± 0.75 ab	1.677 ± 1.677 a	0.160 ± 0.160 a	31.6 ± 1.8 a
	SC+Nano100	38.2 ± 38.2 ab	5.16 ± 0.25 a	5.19 ± 0.31 a	1.459 ± 1.459 b	0.120 ± 0.120 bc	29.8 ± 0.8 ab
	SP+Se5	33.3 ± 33.3 def	3.45 ± 0.16 de	3.80 ± 0.13 d	0.939 ± 0.939 gh	0.078 ± 0.078 e	21.1 ± 1.9 h
	SP+Se10	38.8 ± 38.8 ab	4.83 ± 0.09 a	4.60 ± 0.21 abc	1.311 ± 1.311 cd	0.096 ± 0.096 de	28.7 ± 2.1 bc
	SP+Se20	34.4 ± 34.4 cdef	4.12 ± 0.49 bc	4.14 ± 0.41 bcd	0.902 ± 0.902 hi	0.095 ± 0.095 de	25.6 ± 0.2 de
	SP+Nano5	33.8 ± 33.8 cdef	3.42 ± 0.21 de	3.63 ± 0.12 d	1.054 ± 1.054 efg	0.108 ± 0.108 cd	24.2 ± 0.8 efg
	SP+Nano10	36.6 ± 36.6 abc	3.30 ± 0.11 e	3.71 ± 0.03 d	0.913 ± 0.913 ghi	0.095 ± 0.095 de	23.2 ± 0.1 fgh
	SP+Nano20	33.9 ± 33.9 cdef	3.27 ± 0.24 e	3.90 ± 0.38 d	1.086 ± 1.086 ef	0.096 ± 0.096 de	22.0 ± 0.2 gh
Root	CK	19.7 ± 19.7 f	2.12 ± 0.12 ab	2.00 ± 0.08 ef	0.139 ± 0.139 c	0.882 ± 0.882 def	169.0 ± 11.8 a
	CK + DS	27.2 ± 27.2 e	1.89 ± 0.10 bcd	2.63 ± 0.26 abc	0.124 ± 0.124 cd	0.974 ± 0.974 cdef	110.5 ± 5.9 de
	SC+Se25	37.3 ± 37.3 ab	1.61 ± 0.18 ef	2.78 ± 0.21 a	0.091 ± 0.091 e	0.829 ± 0.829 ef	89.5 ± 9.3 fg
	SC+Se50	33.6 ± 33.6 bcd	2.09 ± 0.16 ab	2.88 ± 0.06 a	0.136 ± 0.136 c	1.132 ± 1.132 cde	117.8 ± 10.9 cd
	SC+Se100	38.1 ± 38.1 a	1.61 ± 0.20 ef	2.78 ± 0.32 a	0.083 ± 0.083 ef	0.810 ± 0.810 f	96.5 ± 7.4 ef
	SC+Nano25	27.1 ± 27.1 e	1.87 ± 0.12 bcd	2.32 ± 0.27 cde	0.105 ± 0.105 de	1.243 ± 1.243 c	90.9 ± 7.8 f
	SC+Nano50	22.4 ± 22.4 f	2.16 ± 0.23 a	2.12 ± 0.32 de	0.173 ± 0.173 b	2.193 ± 2.193 b	137.6 ± 21.7 b
	SC+Nano100	38.4 ± 38.4 a	1.83 ± 0.20 cde	2.90 ± 0.28 a	0.208 ± 0.208 a	2.543 ± 2.543 a	179.7 ± 14.5 a
	SP+Se5	33.0 ± 33.0 cd	1.16 ± 0.04 g	2.30 ± 0.05 de	0.124 ± 0.124 cd	1.169 ± 1.169 cd	69.8 ± 10.6 h
	SP+Se10	36.7 ± 36.7 abc	1.71 ± 0.08 def	2.80 ± 0.02 a	0.136 ± 0.136 c	2.165 ± 2.165 b	128.6 ± 9.2 bc
	SP+Se20	18.6 ± 18.6 f	1.79 ± 0.05 cde	1.77 ± 0.01 f	0.060 ± 0.060 f	0.908 ± 0.908 def	66.3 ± 3.9 h
	SP+Nano5	33.1 ± 33.1 cd	2.03 ± 0.10 abc	2.67 ± 0.17 ab	0.090 ± 0.090 e	2.008 ± 2.008 b	106.4 ± 11.1 def
	SP+Nano10	32.5 ± 32.5 d	1.51 ± 0.09 f	2.44 ± 0.02 bcd	0.104 ± 0.104 de	1.284 ± 1.284 c	72.3 ± 2.6 gh
	SP+Nano20	22.2 ± 22.2 f	1.89 ± 0.23 bcd	1.70 ± 0.09 f	0.093 ± 0.093 e	1.137 ± 1.137 cde	69.7 ± 7.7 h

The data are presented as the mean ± standard deviation (*n* = 4). Different letters indicate significant differences among treatments at *p* < 0.05

Regarding macronutrient accumulation, CK + DS did not differ significantly from CK (Table 3). In seed coating treatments, SC+Se50, SC+Se100, and SC+Nano100 significantly increased K, Ca, and Mg accumulation in both shoots and roots. Among seed-priming treatments, SP+Se5 significantly enhanced macronutrient accumulation in shoots; SP+Se10 significantly increased K and Mg accumulation in whole seedlings and Ca accumulation in shoots; and SP+Nano5 significantly increased K, Ca, and Mg accumulation in all tissues. SP+Nano20 significantly increased the accumulation of these macronutrients in roots.

**Table 3:** Accumulation of the macro and micronutrients in the shoots and roots of 27-day-old rice seedlings after exogenous selenium seed treatment

Tissue	Treatment	K (µg seedling^− 1^)	Ca (µg seedling^− 1^)	Mg (µg seedling^− 1^)	Mn (µg seedling^− 1^)	Fe (µg seedling^− 1^)	Cu (µg seedling^− 1^)
Shoot	CK	2402 ± 85 ghi	273.9 ± 10.2 fg	291.9 ± 14.1 ghi	93.09 ± 4.58 cde	9.58 ± 0.30 cde	2.13 ± 0.06 def
	CK + DS	2608 ± 55 efgh	301.9 ± 14.3 ef	312.1 ± 29.5 fgh	86.36 ± 5.53 e	8.93 ± 0.78 efg	2.08 ± 0.10 def
	SC+Se25	2066 ± 80 hi	242.2 ± 14.8 g	248.3 ± 6.1 i	90.17 ± 5.63 cde	6.95 ± 0.59 h	1.67 ± 0.05 g
	SC+Se50	4249 ± 287 abc	380.1 ± 20.9 bc	417.7 ± 23.5 bc	93.10 ± 5.22 cde	11.07 ± 0.93 bc	2.72 ± 0.12 abc
	SC+Se100	4641 ± 966 a	391.2 ± 74.2 bc	453.6 ± 84.0 b	111.38 ± 23.03 ab	11.55 ± 1.84 ab	2.99 ± 0.61 a
	SC+Nano25	3111 ± 22 de	420.7 ± 11.2 b	423.1 ± 16.6 bc	118.92 ± 1.20 a	9.81 ± 0.19 cde	2.43 ± 0.02 cd
	SC+Nano50	2026 ± 23 i	246.4 ± 26.8 g	269.1 ± 37.5 hi	97.43 ± 4.67 bcde	9.32 ± 0.28 def	1.84 ± 0.07 fg
	SC+Nano100	3769 ± 131 c	358.9 ± 21.0 cd	373.1 ± 20.4 cde	103.53 ± 1.50 bc	12.76 ± 1.79 a	2.37 ± 0.07 cde
	SP+Se5	3175 ± 13 d	328.6 ± 1.2 de	362.4 ± 6.6 def	89.34 ± 1.97 de	7.42 ± 0.13 gh	2.01 ± 0.09 efg
	SP+Se10	4357 ± 220 ab	543.1 ± 29.2 a	516.6 ± 26.4 a	100.97 ± 10.55 bcd	10.61 ± 0.80 bcd	2.88 ± 0.18 ab
	SP+Se20	2518 ± 360 fghi	298.2 ± 20.6 ef	300.1 ± 24.6 ghi	94.87 ± 8.53 cde	6.94 ± 0.60 h	2.09 ± 0.21 def
	SP+Nano5	3964 ± 332 bc	357.7 ± 22.9 cd	402.7 ± 36.0 bcd	99.02 ± 8.17 bcde	10.28 ± 0.73 bcde	2.52 ± 0.23 bc
	SP+Nano10	2977 ± 77 def	301.4 ± 15.3 ef	320.4 ± 15.3 efgh	92.85 ± 4.37 cde	9.44 ± 0.67 def	2.14 ± 0.08 def
	SP+Nano20	2840 ± 385 defg	273.2 ± 32.3 fg	325.0 ± 36.4 efg	90.10 ± 7.60 cde	8.01 ± 1.12 fgh	1.85 ± 0.34 fg
Root	CK	360 ± 11 fg	38.9 ± 1.9 b	36.7 ± 1.7 de	2.55 ± 0.08 defg	16.16 ± 0.69 e	3.09 ± 0.08 a
	CK + DS	497 ± 31 def	34.5 ± 1.2 bc	47.9 ± 2.6 cd	2.26 ± 0.21 gh	17.81 ± 0.80 e	2.02 ± 0.08 e
	SC+Se25	583 ± 82 d	36.2 ± 5.1 bc	50.2 ± 8.4 c	2.36 ± 0.31 efg	19.47 ± 1.60 e	2.03 ± 0.19 e
	SC+Se50	1162 ± 76 ab	50.0 ± 1.5 a	86.7 ± 3.9 a	2.83 ± 0.17 cde	25.77 ± 1.51 d	2.78 ± 0.08 ab
	SC+Se100	1206 ± 283 a	50.8 ± 12.3 a	87.3 ± 16.3 a	2.63 ± 0.58 cdefg	25.57 ± 5.54 d	3.06 ± 0.70 a
	SC+Nano25	541 ± 92 de	37.7 ± 8.2 b	46.1 ± 7.4 cd	3.41 ± 0.41 ab	43.64 ± 7.24 a	2.73 ± 0.42 abc
	SC+Nano50	288 ± 12 g	27.6 ± 0.7 c	27.1 ± 1.9 e	2.65 ± 0.22 cdefg	32.36 ± 3.16 bc	2.30 ± 0.04 cde
	SC+Nano100	1119 ± 71 ab	53.0 ± 2.6 a	84.3 ± 0.2 a	3.05 ± 0.20 bc	36.02 ± 1.76 b	2.64 ± 0.03 bcd
	SP+Se5	1121 ± 10 ab	52.0 ± 1.3 a	85.3 ± 1.3 a	3.79 ± 0.10 a	35.61 ± 2.57 b	2.13 ± 0.27 e
	SP+Se10	1026 ± 144 bc	36.2 ± 5.5 bc	71.6 ± 11.4 b	1.87 ± 0.24 hi	28.17 ± 3.28 cd	2.06 ± 0.26 e
	SP+Se20	314 ± 7 g	30.2 ± 1.1 bc	29.9 ± 1.7 e	2.30 ± 0.15 fgh	36.56 ± 0.97 b	2.17 ± 0.03 e
	SP+Nano5	1035 ± 126 abc	48.4 ± 7.2 a	78.2 ± 12.7 ab	2.95 ± 0.42 bcd	36.21 ± 4.24 b	2.21 ± 0.19 de
	SP+Nano10	394 ± 16 efg	33.6 ± 2.4 bc	30.2 ± 0.9 e	1.60 ± 0.05 i	35.62 ± 2.16 b	1.89 ± 0.04 e
	SP+Nano20	871 ± 77 c	53.7 ± 7.5 a	70.3 ± 8.1 b	2.74 ± 0.32 cdef	33.68 ± 3.41 bc	1.91 ± 0.27 e

The data are presented as the mean ± standard deviation (*n* = 4). Different letters indicate significant differences among treatments at *p* < 0.05

Regarding micronutrient uptake, compared with CK, CK + DS significantly reduced only manganese (Mn) concentration in shoots and copper (Cu) concentration in roots (Table 2). SC+Nano50 and SC+Nano100 significantly increased Mn and Cu concentrations in seedling tissues and iron (Fe) concentration in roots. SP+Se10 significantly increased Mn concentration in shoots and Fe and Cu concentrations in roots, while other elements showed no significant differences from CK + DS. SC+Se50 significantly increased Mn concentration in shoots, whereas concentrations of other micronutrients were comparable to or slightly higher than CK + DS but without significance.

Regarding micronutrient accumulation, CK + DS significantly reduced only root Cu accumulation, with no significant differences for other elements compared with CK (Table 3). Among seed coating treatments, SC+Se50 and SC+Se100 significantly enhanced Mn accumulation in roots and shoots, respectively, and both increased Fe and Cu accumulation in plant tissues. SC+Nano25 significantly increased total micronutrient accumulation in roots, while SC+Nano100 significantly enhanced micronutrient accumulation in all seedling tissues. In seed-priming treatments, SP+Se10 significantly increased total micronutrient accumulation in shoots and Fe accumulation in roots, with no significant effects on other elements relative to CK + DS. SP+Nano5 significantly increased Cu accumulation in shoots and Mn and Fe accumulation in roots, while other micronutrient accumulations showed slight but non-significant increases compared with CK + DS.

### Effects of seed treatment with Se on element translocation from roots to shoots

Compared with normal moisture conditions, drought stress reduced the Se TF, although the reduction was not significant (*P* > 0.05, Table 4). Appropriate exogenous selenium treatments markedly enhanced Se TF under drought conditions. Relative to CK + DS, SC+Se50, SP+Se10, SP+Nano5, and SP+Nano20 increased Se TF by 24.6%, 21.5%, 17.2%, and 16.2%, respectively, with no significant differences among these treatments. For heavy metal translocation, drought stress significantly increased the TFs of Cr and Pb, while significantly decreasing that of Cd (Table 4). Drought had no significant effect on As TF (*P* > 0.05). For Cr TF, all exogenous selenium treatments except SC+Nano50 significantly reduced its value, with SC+Se100, SP+Se5, and SP+Nano20 showing the strongest reductions. For As TF, all Nano-Se treatments and SP+Se20 significantly decreased its value, except SP+Nano5, which showed no significant effect. For Cd TF, all exogenous selenium treatments increased its value to varying degrees; SC+Se50 and SP+Nano5 were the only treatments not significantly different from CK + DS. For Pb TF, all exogenous selenium treatments except SP+Se10 significantly reduced its value, with SP+Se20 and SP+Nano10 showing the greatest reductions.

**Table 4:** Effects of seed treatment with Se on the transfer factor of Se, Cr, As, Cd, and Pb in the rice seedlings

Treatment	Se	Cr	As	Cd	Pb
CK	0.713 ± 0.083 bcd	0.389 ± 0.038 de	0.086 ± 0.011 def	0.193 ± 0.013 def	0.064 ± 0.003 d
CK + DS	0.679 ± 0.068 d	1.055 ± 0.034 a	0.091 ± 0.002 cde	0.148 ± 0.026 g	0.087 ± 0.001 b
SC+Se25	0.649 ± 0.029 d	0.272 ± 0.016 fg	0.082 ± 0.011 efg	0.172 ± 0.011 efg	0.035 ± 0.004 g
SC+Se50	0.846 ± 0.077 a	0.350 ± 0.020 ef	0.105 ± 0.016 c	0.153 ± 0.007 fg	0.062 ± 0.005 d
SC+Se100	0.748 ± 0.101 abcd	0.208 ± 0.019 g	0.229 ± 0.014 a	0.251 ± 0.035 ab	0.051 ± 0.007 f
SC+Nano25	0.485 ± 0.061 e	0.608 ± 0.020 b	0.072 ± 0.012 fgh	0.256 ± 0.035 a	0.052 ± 0.002 ef
SC+Nano50	0.690 ± 0.032 cd	1.151 ± 0.190 a	0.065 ± 0.004 gh	0.159 ± 0.028 fg	0.033 ± 0.006 g
SC+Nano100	0.643 ± 0.035 d	0.513 ± 0.051 bc	0.060 ± 0.009 h	0.220 ± 0.029 abcd	0.036 ± 0.006 g
SP+Se5	0.653 ± 0.020 d	0.220 ± 0.030 g	0.142 ± 0.026 b	0.234 ± 0.013 abc	0.078 ± 0.001 c
SP+Se10	0.825 ± 0.085 a	0.534 ± 0.110 bc	0.103 ± 0.003 cd	0.200 ± 0.036 cde	0.102 ± 0.006 a
SP+Se20	0.429 ± 0.009 e	0.378 ± 0.043 def	0.072 ± 0.006 fgh	0.169 ± 0.012 efg	0.024 ± 0.003 h
SP+Nano5	0.796 ± 0.095 ab	0.463 ± 0.030 cd	0.078 ± 0.009 efgh	0.151 ± 0.021 g	0.033 ± 0.001 g
SP+Nano10	0.409 ± 0.042 e	0.433 ± 0.018 cde	0.019 ± 0.002 i	0.160 ± 0.027 efg	0.029 ± 0.005 gh
SP+Nano20	0.789 ± 0.051 abc	0.223 ± 0.009 g	0.064 ± 0.010 gh	0.215 ± 0.013 bcd	0.059 ± 0.004 de

The data are presented as the mean ± standard deviation (*n* = 4). Different letters indicate significant differences among treatments at *p* < 0.05

## Discussion

Drought stress significantly suppressed the growth and development, photosynthetic performance, and mineral nutrient balance of rice seedlings. This finding is highly consistent with existing research indicating that water deficit leads to restricted carbon assimilation and disrupted ion homeostasis [35–37]. Building upon this, the present study further revealed that applying selenium in suitable forms and doses via seed priming or seed coating can synergistically mitigate the adverse effects of drought stress on rice across multiple physiological levels. However, this regulatory effect exhibits distinct selenium source dependency and application method specificity. These differences primarily stem from the fundamental variations between ionic selenium and nano-selenium in absorption kinetics, bioavailability, and intracellular regulatory pathways, leading to markedly different mechanisms of action in mitigating drought stress.

In terms of plant growth responses, both selenite and nano-selenium exhibited pronounced dose-dependent effects under different application methods, though their optimal concentration ranges and biological response stability showed significant differences. Overall, selenite exhibited a typical medium-concentration optimal dose effect in both seed coating and priming treatments: under coating conditions, the medium-concentration treatment (SC+Se50) most significantly promoted seedling growth, whereas in seed priming, the optimal concentration was markedly lower (SP+Se10). This discrepancy likely stems from differing selenium release rates and initial exposure intensities between the two application methods. Seed coating typically provides sustained selenium release during germination and early growth stages, requiring relatively high application levels to maintain effective supply. In contrast, priming treatment induces a short-term yet highly efficient stimulatory effect during early germination, where physiological responses can be triggered by low doses. Previous studies have also demonstrated that low-dose selenium enhances seedling tolerance to drought stress through preactivation mechanisms, whereas excessive selenium may induce oxidative stress or ion imbalance, thereby counteracting its beneficial effects [10, 38]. In contrast, nano-selenium exhibits a broader safety window and more stable growth-promoting effects, with dose-response characteristics markedly different from those of selenite. Particularly in seed-priming treatments, low concentrations of nano-selenium (SP+Nano5) significantly promoted seedling growth, whereas its stimulatory effect tended to diminish with increasing application concentrations. This phenomenon indicates that nano-selenium exhibits high bioactivity during early germination stages, with its effects not solely dependent on application rate but more likely linked to its efficient absorption and controlled-release properties during the radicle formation phase. In contrast, under seed coating conditions, nano-selenium requires higher application levels (SC+Nano100) to fully exert its promoting effect. This suggests that the physical barrier within the coating system may partially limit the release and utilization efficiency of nano-selenium.

Changes in photosynthetic performance provide direct physiological evidence that selenium alleviates drought stress. Drought stress simultaneously reduced *A*, *g*_*sw*_, and *E* in rice leaves, indicating that stomatal limitation is the primary factor restricting photosynthesis under water deficit [39]. In this study, exogenous selenium treatments such as SC+Se50, SC+Nano100, and SP+Nano5 significantly increased both *A* and *g*_*sw*_, exhibiting consistent patterns. These results confirm that appropriate selenium treatment alleviates drought-induced photosynthetic inhibition by enhancing CO₂ supply through improved stomatal regulation. Furthermore, the extent of improvement in net photosynthetic rate and stomatal conductance under different selenium treatments largely aligns with existing research findings on selenium-mediated alleviation of abiotic stress [40]. This further supports the notion that selenium enhances plant drought tolerance by maintaining stomatal function and carbon assimilation capacity. Notably, SC+Nano100 and SP+Nano5 not only increased *A* but also significantly elevated *Ci*. This pattern exceeds the scope of simple stomatal limitation and suggests that Nano-Se may also exert non-stomatal protective effects on the photosynthetic apparatus. From a molecular mechanism perspective, this non-stomatal effect may be related to the enhancement of the antioxidant defense system by Nano-Se. Specifically, selenium nanoparticles can increase the activity of key enzymes such as superoxide dismutase (SOD), catalase (CAT), and ascorbate peroxidase (APX), thereby reducing the accumulation of ROS in chloroplasts and alleviating oxidative damage. This process helps maintain the structural stability of photosystem II and the activity of the Rubisco carboxylase, thereby improving photosynthetic efficiency and carbon assimilation capacity in addition to enhancing stomatal conductance. This also provides a reasonable explanation for the simultaneous increase in *Ci* and *A* observed in this study. Combined with the significant increase in leaf SPAD values, it is inferred that nanoselenium improves photosystem function and carboxylation efficiency by alleviating oxidative stress and maintaining chloroplast structural integrity [18]. This inference aligns with existing reports indicating that nano-selenium enhances plant antioxidant defense systems under stress conditions [14, 41]. However, the specific molecular regulatory mechanisms underlying this effect within the present study system require further in-depth analysis.

The ability of plants to survive drought stress largely depends on the water-acquisition efficiency of their root systems. This study demonstrates that appropriate exogenous selenium treatments substantially alleviate drought-induced inhibition of root growth by promoting the formation of a more developed root system characterized by greater total length, surface area, and volume. Previous studies have indicated that selenium promotes root growth and development by regulating the expression of genes associated with root cell division and elongation, while reducing damage to root apical meristems caused by ROS [42]. The resulting optimized root architecture is considered a crucial structural foundation for enhancing plant drought tolerance [43]. This optimized root architecture facilitates expanded soil exploration, enhances water uptake efficiency, and ultimately manifests as a significant increase in relative leaf water content, with the SP+Nano5 treatment demonstrating the most pronounced effect. Notably, despite its superior root morphological traits, SP+Nano5 resulted in significantly lower aboveground fresh weight compared with SC+Se50 and SP+Se10. This seemingly contradictory outcome reflects complex resource allocation strategies under drought: constructing an extensive root system requires considerable photosynthates and metabolic energy [44]. Thus, SP+Nano5 likely prioritized belowground investment to establish a robust water-absorption network, temporarily constraining shoot biomass accumulation but securing long-term water supply and physiological stability. Overall, seed priming generally outperformed seed coating in enhancing root development. This advantage may arise because priming allows selenium to participate directly in early root initiation and differentiation during germination, thereby establishing a more favorable intrinsic root architecture [45]. Moreover, nano-selenium effectively promoted root growth even at low concentrations, further confirming its low phytotoxicity and high bioactivity. This avoids the inhibition of root-tip cell division and elongation that can occur when excessive ionic selenium induces oxidative damage [46]. In summary, exogenous selenium—particularly nano-selenium applied via seed priming—fundamentally strengthens plant water-acquisition capacity by prioritizing root system regulation, thereby providing a solid physiological foundation for the recovery and maintenance of aboveground growth and metabolism.

The dynamic distribution and accumulation patterns of selenium within plants under different selenium treatments elucidate the mechanistic basis for the form- and application-specific effects observed in this study. Selenium content in plant tissues generally exhibited a concentration-dependent response to selenite, characterized by an initial increase followed by a decline at higher concentrations. In treatments such as SC+Se100 and SP+Se20, both selenium accumulation and plant growth were inhibited, indicating that ionic selenium (Se(IV)) becomes phytotoxic beyond a certain threshold [12]. This toxicity may arise from rapid intracellular reduction of Se(IV), leading to excessive generation of ROS, or from disruption of essential physiological processes, including sulfur metabolism [6]. In contrast, nano-selenium treatments—particularly under seed coating—showed a concentration-dependent enhancement of selenium uptake and accumulation, with SC+Nano100 demonstrating the strongest effect. This pattern highlights the inherently low toxicity and high biocompatibility of nano-selenium, which is often attributed to its controlled-release behavior and gradual redox transformation within plant tissues [20]. Under seed-priming conditions, even extremely low concentrations of nano-selenium (e.g., SP+Nano5) facilitated efficient selenium accumulation. This suggests that nanoparticles may enter root or embryonic tissues through non-ionic pathways such as endocytosis during early germination and subsequently serve as a stable, low-toxicity selenium reservoir within the plant [47]. At the molecular level, the absorption and transport of selenium may involve the coordinated regulation of sulfur transport systems (such as proteins of the SULTR family) and phosphate transporters [48]. Due to its particulate nature, Nano-Se may partially bypass conventional ion channels, entering cells via endocytosis and gradually converting into metabolizable forms within the body, thereby achieving low-toxicity and highly efficient utilization. Importantly, the effective selenium treatments identified in this study (SC+Se50, SP+Se10, SP+Nano5) significantly increased the selenium transport factor from roots to shoots. This indicates that exogenous selenium not only enhances the root system’s ability to absorb selenium but also effectively promotes its long-distance transport within the plant. This process may be closely related to the regulation of selenium-related transporter expression levels [49]. This finding aligns with existing research indicating that selenium enhances nutrient distribution efficiency by regulating nutrient transport systems [50]. Previous studies have demonstrated that selenium modulates its translocation and distribution within plants by influencing sulfur assimilation pathways and the expression of associated transporters [51].

At the ion homeostasis level, our findings clearly demonstrate that selenium exerts dual regulatory effects in alleviating drought stress: it inhibits the uptake and upward transport of toxic elements while promoting the absorption and accumulation of essential mineral elements. Drought stress markedly increased the accumulation of Cr, As, Cd, and Pb in rice seedlings, likely due to impaired root selective absorption barriers and the activation of nonspecific ion channels that facilitate uncontrolled metal influx. However, appropriate selenium treatments—particularly SC+Se50, SC+Nano100, SP+Se10, and SP+Nano5—effectively counteracted this trend by significantly reducing both the concentrations and total accumulation of these toxic elements in aboveground tissues. Several mechanisms may underlie this antagonistic effect. First, selenium (especially Se(IV)) may compete with heavy metal ions for shared transport pathways at the root–soil interface, including phosphate transporters or divalent metal ion transporters [12]. For example, Se(IV) can enter plants via phosphate transporters (such as OsPT8), thereby competitively inhibiting the uptake of anions such as As(V) at the absorption site [52]; simultaneously, certain heavy metals (such as Cd²⁺) may enter cells via IRT-type transporters, and selenium treatment can reduce their permeability by regulating the expression levels of these transporters [53]. Second, selenium may stimulate the synthesis of endogenous chelators or enhance heavy metal immobilization within root cell walls, thereby promoting sequestration and retention in root tissues. Previous studies have shown that selenium can induce the synthesis of glutathione and plant chelating peptides, and promote the formation of stable complexes between heavy metals and these ligands, thereby limiting their transport to the aboveground parts [54]. Furthermore, selenium may also improve the efficiency of heavy metal immobilization in roots by enhancing the metal-binding capacity of pectin and hemicellulose in the cell wall [55]. This restricted mobility is supported by reduced heavy metal transport factors (TFs) observed under effective treatments, indicating suppression of long-distance translocation from roots to shoots [56].

Importantly, selenium’s antagonism toward heavy metals exhibits pronounced form- and concentration-dependent specificity. Low to moderate selenite levels (e.g., SC+Se50, SP+Se10) exert strong detoxifying effects, whereas high concentrations (e.g., SC+Se100, SP+Se20) paradoxically increase the accumulation of Cr, As, or Pb in roots or elevate As concentrations in shoots. It should be noted that in this study, some treatments exhibited increased heavy metal concentrations in the roots while showing decreased concentrations in the shoots. This is not a contradiction but rather a typical detoxification strategy employed by plants in response to heavy metal stress: by enhancing the fixation, chelation, or cell wall adsorption of heavy metals in the roots, the plants limit their transport to the aboveground parts, thereby protecting photosynthetic tissues from toxicity. This explanation is consistent with the reduced heavy metal transport coefficients observed in the experiment. This dual effect of low-concentration promotion and high-concentration inhibition may stem from oxidative stress triggered by excessive Se(IV), which disrupts intracellular redox balance and membrane integrity, thereby weakening ion-selective mechanisms and increasing passive heavy metal influx [57]. This finding aligns with previous reports that high selenium levels alter root permeability and promote heavy metal retention in roots [58]. In contrast, nano-selenium treatments, such as SC+Nano100, consistently suppressed heavy metal accumulation in aboveground tissues while simultaneously promoting plant growth and selenium uptake, without inducing synergistic toxicity. This advantage may be related to the unique physicochemical properties of Nano-Se and its sustained-release behavior within plants. On the one hand, despite its small particle size, Nano-Se has a low dissolution rate, allowing it to gradually release available selenium in the rhizosphere environment, thereby avoiding toxic effects caused by transient high concentrations; on the other hand, Nano-Se may enter cells via aquaporin-mediated pathways, with an absorption route distinct from that of conventional inorganic selenium, thereby reducing direct interference with the ion channel system [59]. Furthermore, Nano-Se can more efficiently activate the antioxidant defense system, maintain cellular redox homeostasis, and indirectly enhance membrane structural stability and ion-selective absorption capacity. These findings highlight nano-selenium’s potential as a safe and effective selenium source capable of achieving dual functional goals: enhancing plant selenium nutrition and mitigating heavy metal contamination risks.

Meanwhile, selenium treatment significantly promoted the uptake and accumulation of essential mineral elements, indicating that selenium’s role extends beyond indirectly improving plant growth by reducing toxicity burdens—it systematically enhances plants’ ability to acquire mineral nutrients. In this study, treatments such as SC+Se50, SC+Nano100, SP+Se10, and SP+Nano5 not only mitigated drought-induced growth inhibition but also significantly increased both the concentrations and total accumulation of key macronutrients (K, Ca, Mg) and micronutrients (Mn, Cu, Fe) in aboveground and root tissues. K, Ca, and Mg play irreplaceable roles in regulating osmotic potential, maintaining enzyme activity, and stabilizing membrane structure [60]. Their increased content helps maintain cellular water balance and metabolic activity, forming a crucial physiological foundation for plant drought response [61, 62]. Concurrently, increased levels of trace elements like Mn, Fe, and Cu may relate to selenium-induced activation of antioxidant enzyme systems, as these elements are essential components of multiple antioxidant enzymes. Studies confirm selenium enhances plant antioxidant capacity and promotes optimal trace element distribution within the plant body [63]. From a mechanistic perspective, the observed nutrient enhancement may be attributed to multiple selenium-mediated mechanisms. First, selenium-induced optimization of root architecture likely expanded the effective absorption surface area, thereby improving nutrient acquisition under limited water availability. More critically, growing evidence indicates that selenium may directly modulate mineral nutrient metabolism by regulating the expression of genes involved in nutrient transport, homeostasis, and compartmentalization [64]. Additionally, owing to its chemical similarity to sulfur, selenium absorption and assimilation are intertwined with sulfur metabolic pathways. This interaction may influence the biosynthesis of sulfur-containing amino acids and associated metabolites, thereby indirectly affecting the integration, distribution, and transport of other essential elements [65]. Therefore, the enhancement of mineral nutrient levels observed in this study is not merely a result of growth dilution effects, but more likely reflects the combined outcome of selenium’s ability to strengthen the plant’s overall ion homeostasis regulation and coordinate nutrient metabolism.

## Conclusion

This study demonstrated that nano-selenium is substantially more effective than traditional selenite in enhancing rice drought tolerance. Low-concentration nano-selenium applied through seed priming simultaneously improved drought resistance, strengthened selenium nutrition, and reduced heavy metal accumulation. Its protective effects were associated with multiple coordinated mechanisms, including: (i) alleviating plant toxicity through sustained-release behavior; (ii) improving water uptake efficiency by optimizing root system architecture; (iii) maintaining photosynthetic capacity by enhancing stomatal conductance; and (iv) promoting the absorption of essential nutrients while suppressing the upward transport of heavy metals via synergistic regulation of ion balance. Together, these mechanisms collectively and systematically enhanced plant drought adaptability. This research provides a theoretical foundation for developing efficient and low-risk selenium-based drought-mitigation formulations, carrying important practical value for safe grain production and nutritional improvement in heavy metal-contaminated regions. Nevertheless, the molecular mechanisms through which selenium regulates heavy metal transporters remain insufficiently understood, and findings based on hydroponic experiments require further validation under field conditions. Future studies integrating multi-omics approaches with long-term field trials will be crucial for elucidating selenium’s regulatory network and assessing its agronomic applicability.

## Data Availability

The datasets used and/or analysed during the current study are available from the corresponding author on reasonable request.
